# Pharmacological Effects and Proposed Mechanisms of *Atractylodes* Medicinal Plants in Liver Diseases: A Narrative Review

**DOI:** 10.3390/life16091476

**Published:** 2026-09-03

**Authors:** Jin Sun, Rumeng Wei, Xinyu Wang, Miaomiao Gao, Shuai Cao, Xiangsong Meng, Juhui Qiao

**Affiliations:** School of Chinese Medicine, Bozhou University, Bozhou 236800, China; sunjin0509@163.com (J.S.);

**Keywords:** *Atractylodes*, liver diseases, alcohol-associated liver disease (ALD), non-alcoholic fatty liver disease (NAFLD), metabolic dysfunction-associated steatotic liver disease (MASLD), gut–liver axis

## Abstract

Medicinal plants of the genus *Atractylodes*, primarily including *Atractylodes lancea* (Thunb.) DC., *Atractylodes chinensis* (DC.) Koidz., and *Atractylodes macrocephala* Koidz., are important traditional Chinese medicinal herbs that are widely recognized for their functions of “strengthening the spleen and drying dampness” and have traditionally been used to treat digestive and metabolic disorders. In recent years, with the increasing global burden of liver diseases, *Atractylodes* species have attracted growing attention for their potential pharmacological value in liver disease management due to their abundant bioactive compounds and diverse pharmacological activities. This review summarizes reported biological and hepatoprotective effects and proposed mechanisms of *Atractylodes* medicinal plants and their bioactive compounds in liver-related experimental models, including models relevant to MASLD, ALD, hepatic fibrosis, and HCC. Preclinical studies have reported changes in lipid metabolism, oxidative-stress-related markers, inflammatory responses, fibrotic indices, and tumor-cell phenotypes, accompanied by modulation of signaling pathways including AMPK/SIRT1, PPAR, Nrf2/HO-1, TLR4/MyD88/NF-κB, NLRP3, and TGF-β1/Smad. However, much of the mechanistic evidence is based on pathway-associated changes rather than direct compound–target validation, and clinical evidence remains limited. Overall, the available findings provide a preclinical pharmacological rationale for further investigation of *Atractylodes*-derived compounds in liver diseases, while their clinical efficacy and therapeutic roles remain to be established.

## 1. Introduction

Chronic liver diseases comprise etiologically distinct disorders, including metabolic dysfunction-associated steatotic liver disease (MASLD) and alcohol-associated liver disease (ALD), that may share pathological processes such as steatosis, inflammation, and fibrosis. Persistent liver injury can lead to advanced structural consequences such as cirrhosis, while hepatocellular carcinoma (HCC) may develop as a malignant complication in some chronic liver disease settings [1,2]. Current management strategies for liver disease include lifestyle interventions, metabolic modulators, anti-inflammatory drugs, and targeted therapies, which have improved clinical outcomes for many patients with MASLD, ALD, liver fibrosis, and HCC. However, challenges remain, including interindividual differences in treatment efficacy, adverse reactions associated with long-term use of certain medications, and limited treatment options for patients with advanced or refractory disease [3,4]. These unmet clinical needs continue to drive researchers to explore complementary or adjunctive therapeutic strategies. Against this backdrop, traditional medicinal plants, with their multi-component and multi-target characteristics, have become a unique resource for identifying potential candidate compounds and therapeutic approaches, with *Atractylodes* species receiving particular attention [5].

The genus *Atractylodes* (Asteraceae) comprises approximately eight species, primarily distributed in East Asia [6]. Among these, *Atractylodes lancea* (Thunb.) DC. and *Atractylodes chinensis* (DC.) Koidz. are recognized in the Chinese, Japanese, and Korean pharmacopoeias as the botanical sources of the crude drug “Cangzhu” (Japanese: Sojutsu; Korean: Changchul), and are collectively referred to as Atractylodis Rhizoma (AR) in this review. *Atractylodes macrocephala* Koidz. is recognized in these pharmacopoeias as the botanical source of “Baizhu” (Japanese: Byakujutsu; Korean: Baekchul), and is referred to as Atractylodis Macrocephalae Rhizoma (AMR) in this review. Both have their respective emphases in the clinical application of traditional Chinese medicine: AR is traditionally used for drying dampness, strengthening the spleen, dispelling wind-cold, and treating abdominal distension, diarrhea, and rheumatic pain caused by dampness blocking the middle energizer; AMR tends to invigorate qi and the spleen, dry dampness, and promote diuresis, making it more suitable for poor appetite, fatigue, edema, phlegm-fluid retention, and fetal restlessness caused by spleen deficiency and dampness [6,7]. Although their therapeutic focuses differ, both share the core function of invigorating the spleen and removing dampness. This traditional functional orientation may be interpreted in the context of the pathogenesis chain of modern liver disease, which encompasses metabolic disorder, inflammatory injury, and microecological imbalance.

The chemical constituents contained in AR and AMR are both overlapping yet distinct, which together constitute the material basis of their liver protective activity. The common components of the two kinds of medicinal materials include atractylon, β-eudesmol and small amounts of other sesquiterpenes. However, the species-specific chemical characteristics are more distinct: AR uses polyacetylenes such as atractylodin and atractylodinol as its specific marker components (atractylodin is used as a quality control index in Chinese Pharmacopoeia), while AMR uses sesquiterpene lactones such as atractylenolide I, II, and III as its core active group (atractylenolide I is an important quality control component of AMR) [5,8]. In addition, both AR and AMR contain polysaccharide constituents that have been reported to exhibit immunomodulatory activity in experimental settings. This compositional profile contributes to the distinct pharmacological characteristics of AR and AMR. Atractylodin has been associated with modulation of oxidative stress and ferroptosis, whereas atractylenolides have been reported to affect hepatic fibrosis, HSC senescence, and autophagy in experimental models [9,10]. Collectively, the available evidence indicates that AR and AMR contain distinct sets of bioactive constituents with partially overlapping and partially complementary pharmacological properties in preclinical models. These compositional and functional features support the continued investigation of *Atractylodes* species as a source of multiple bioactive compounds relevant to liver disease research.

In recent years, with the deep integration of network pharmacology, metabolomics, molecular biology and intestinal microecology, the pharmacological mechanisms of *Atractylodes* in liver diseases have been gradually elucidated. Studies have reported that AR- and AMR-derived active ingredients are associated with the modulation of key signaling pathways related to various liver diseases: in the field of metabolism, observed AMPK activation correlates with reduced de novo lipid synthesis and enhanced fatty acid oxidation [11]; in the field of inflammation, suppression of the TLR4/MyD88/NF-κB signaling axis has been observed in parallel with reduced “intestinal endotoxin-liver inflammation” cascade damage [12]; in the field of stress, enhanced Nrf2/HO-1 antioxidant responses and inhibition of ferroptosis-related markers have been reported to coincide with improved redox balance in hepatocytes [9]; in the field of fibrosis, downregulation of TGF-β1/Smad signaling and induction of hepatic stellate cell senescence are associated with reduced extracellular matrix deposition [13]; in the field of carcinogenesis, activation of the mitochondrial apoptosis pathway and suppression of EMT/MMP-mediated invasion have been observed, suggesting potential to inhibit the progression of liver cancer [14].

Based on the aforementioned research advances, this paper aims to systematically review the mechanisms of action of the major bioactive components in *Atractylodes* (AR and AMR) medicinal materials for various liver diseases, focusing on the following aspects: the regulatory effects of bioactive components on metabolism, inflammation, oxidative stress, fibrosis, and hepatocellular carcinoma; and systemic intervention models such as regulation of the gut–liver axis. By integrating representative findings from recent studies, this review aims to establish a comprehensive mechanistic framework for the hepatoprotective effects of *Atractylodes* species, to assess their translational potential, and to identify key knowledge gaps and future research priorities, thereby providing a reference for the rational development and evidence-based application of *Atractylodes* in liver disease management.

## 2. Methods

### 2.1. Search Strategy

A literature search was conducted using PubMed, Web of Science, Scopus, Google Scholar, the Cochrane Library, and China National Knowledge Infrastructure (CNKI), covering the period from 1996 to 2026 and limited to articles in Chinese and English. The search strategy focused on two medicinal materials: AR and AMR. Keywords were categorized into five groups based on their logical relationships: (1) species names (e.g., “*Atractylodes lancea*,” “*Atractylodes chinensis*,” “*Atractylodes macrocephala*,” “Cangzhu,” “Baizhu”); (2) extracts and chemical constituents (e.g., “volatile oil,” “polysaccharides,” “atractylodin,” “atractylon,” “atractylenolide”); (3) pharmacological activities (e.g., “hepatoprotective,” “anti-inflammatory,” “antioxidant”); (4) types of liver diseases (e.g., “liver diseases,” “ALD,” “MASLD,” “liver fibrosis,” “hepatocellular carcinoma”); (5) mechanism-related areas (e.g., “gut–liver axis,” “oxidative stress,” “inflammatory signaling”). Two researchers independently conducted an initial screening of titles and abstracts and a subsequent full-text review of all literature. For review articles, the references were traced to identify any omitted original studies, and a manual search was conducted to supplement key literature. Following the evaluation, a total of 150 original research studies and high-quality reviews were ultimately included.

### 2.2. Positioning of the Present Review

Unlike previous reviews that primarily focused on a single *Atractylodes* species or a single type of liver disease, the present review achieves systematic integration at three distinct levels: (1) covering the three major species of the genus *Atractylodes*—*A. lancea*/*A. chinensis* (AR) and *A. macrocephala* (AMR); (2) integrating evidence across etiologically defined liver diseases (MASLD and ALD), shared pathological processes and advanced consequences of chronic liver injury (fibrosis and cirrhosis), and HCC; and (3) critically evaluating pharmacokinetic, toxicological, and translational limitations. To clearly define the breakthroughs of this review relative to previous research, the following section compares the main features of this review with those of representative previous reviews (Table 1).

### 2.3. Figure Preparation

The figures in this article were created using a variety of software tools, including Home for Researchers (https://professional.home-for-researchers.com/, accessed on 15 August 2026), Microsoft PowerPoint 2024 (https://www.microsoft.com/) and Adobe Photoshop 2025.

## 3. Characteristics of Major Chemical Constituents in *Atractylodes* Medicinal Plants

The active components of *Atractylodes* species are dominated by volatile oils, which include terpenoids (mainly sesquiterpenes), polyacetylenes, and aromatic compounds [17]. A clear species-dependent chemical differentiation exists: the volatile oils of *A. lancea* and *A. chinensis* are characterized by high levels of atractylodin, whereas the volatile oil of *A. macrocephala* is dominated by atractylon, which can account for more than 60% of the total volatile oil content [18,19]. In terms of non-volatile components, all three species contain organic acids and glycosides, though their specific profiles differ [6,20]. Regarding quality control, the Pharmacopoeia stipulates that atractylodin content must not be less than 0.15% for *A. lancea* and 0.30% for *A. chinensis*, while atractylodin, β-eudesmol, and atractylon are commonly used as quality indicators. For *A. macrocephala*, although only the alcohol-soluble extract content is specified as a statutory requirement, atractylon and atractylenolides I–III are frequently employed as quality indicators in research practice. Quantitative comparisons reveal a clear gradient: the total volatile oil, atractylodin, and β-eudesmol contents decrease progressively from *A. lancea* to *A. chinensis* to *A. macrocephala*, whereas atractylon content shows the opposite trend [21,22,23,24].

Geographical and ecological factors significantly influence the accumulation of chemical constituents. *A. lancea* is predominantly cultivated in warmer, humid regions of southern China (e.g., Jiangsu, Hubei), conditions conducive to higher volatile oil accumulation, whereas *A. chinensis* thrives in the drier, colder regions of northern China (e.g., Hebei, Shanxi, Inner Mongolia) with correspondingly lower volatile oil content [25,26]. *A. macrocephala* from different producing areas shows volatile oil contents ranging from 0.58% to 1.22%, with atractylon consistently as the most abundant component, although the specific volatile profiles differ across regions [27]. These origin-dependent chemical differences provide a material basis for evaluating authenticity and guide subsequent quality control and mechanistic studies. Notably, the contents of bioactive constituents in *Atractylodes* species vary with plant age and harvesting season. Studies have shown that the levels of constituents such as atractylodin and β-eudesmol in AR fluctuate significantly across different harvesting periods, highlighting the importance of harvesting at an appropriate time to ensure the quality of the medicinal material [28,29].

Furthermore, processing can significantly alter the chemical composition of medicinal materials derived from *Atractylodes* species. Studies have demonstrated marked differences in both volatile and nonvolatile chemical profiles between raw and bran-fried AR, and the content of atractylodin is closely associated with the degree of processing, suggesting its potential as an important marker for quality evaluation during bran-frying [30,31]. A recent GC-MS study further identified 95 constituents in raw and bran-fried samples and showed that bran-frying markedly altered the volatile chemical profile, with substantial decreases in constituents such as elemol [32]. These findings indicate that heating and the use of processing adjuvants may induce the volatilization, degradation, or transformation of terpenoid constituents. AMR also undergoes pronounced chemical transformations during stir-frying, characterized by decreases in volatile oil and atractylon and increases in atractylenolides I, II, and III; some of these changes may be attributable to heat-induced conversion of atractylon into lactone derivatives [33,34]. In addition, stir-frying can modify the structural and functional properties of AMR polysaccharides [35]. Therefore, processing represents an important factor affecting the bioactive material basis and quality consistency of both AR and AMR.

Collectively, these findings indicate that, in pharmacological studies and clinical applications of *Atractylodes*-derived medicinal materials, the effects of geographical origin, harvesting period, and processing methods on the levels of bioactive constituents should be fully considered. Rigorous quality-control standards should therefore be established to ensure the comparability of research findings and the consistency of clinical efficacy.

## 4. Traditional Uses of *Atractylodes* and Possible Relationships with Liver-Related Biological Processes

In traditional Chinese medicine (TCM), *Atractylodes* medicinal plants are primarily characterized by the therapeutic principle of “invigorating the spleen and drying dampness” [6]. According to TCM theory, the spleen-stomach system is responsible for the transformation and transportation of nutrients and plays an essential role in maintaining overall physiological homeostasis [36]. In addition, the concept of “liver-spleen coordination” emphasizes the importance of their interaction in regulating the circulation of qi and blood, metabolic balance, and organ function [37]. Dysfunction of spleen transportation and transformation is considered to contribute to the accumulation of endogenous dampness and subsequent impairment of liver function [38]. From a modern biomedical perspective, this concept has been linked to several pathological processes relevant to liver diseases, including lipid metabolism disorders, chronic inflammation, oxidative stress, and impaired energy metabolism [39,40]. Increasing evidence demonstrates that *Atractylodes*-derived bioactive constituents exert multiple hepatoprotective effects, including improvement of abnormal lipid metabolism, attenuation of oxidative damage, inhibition of inflammatory responses, suppression of fibrosis progression, reduction in serum transaminase levels, and regulation of bile metabolism. These findings suggest the potential therapeutic value of *Atractylodes* medicinal plants in various liver disease models, including fatty liver disease, chronic hepatitis, and hepatic fibrosis [10].

**Figure 1:**
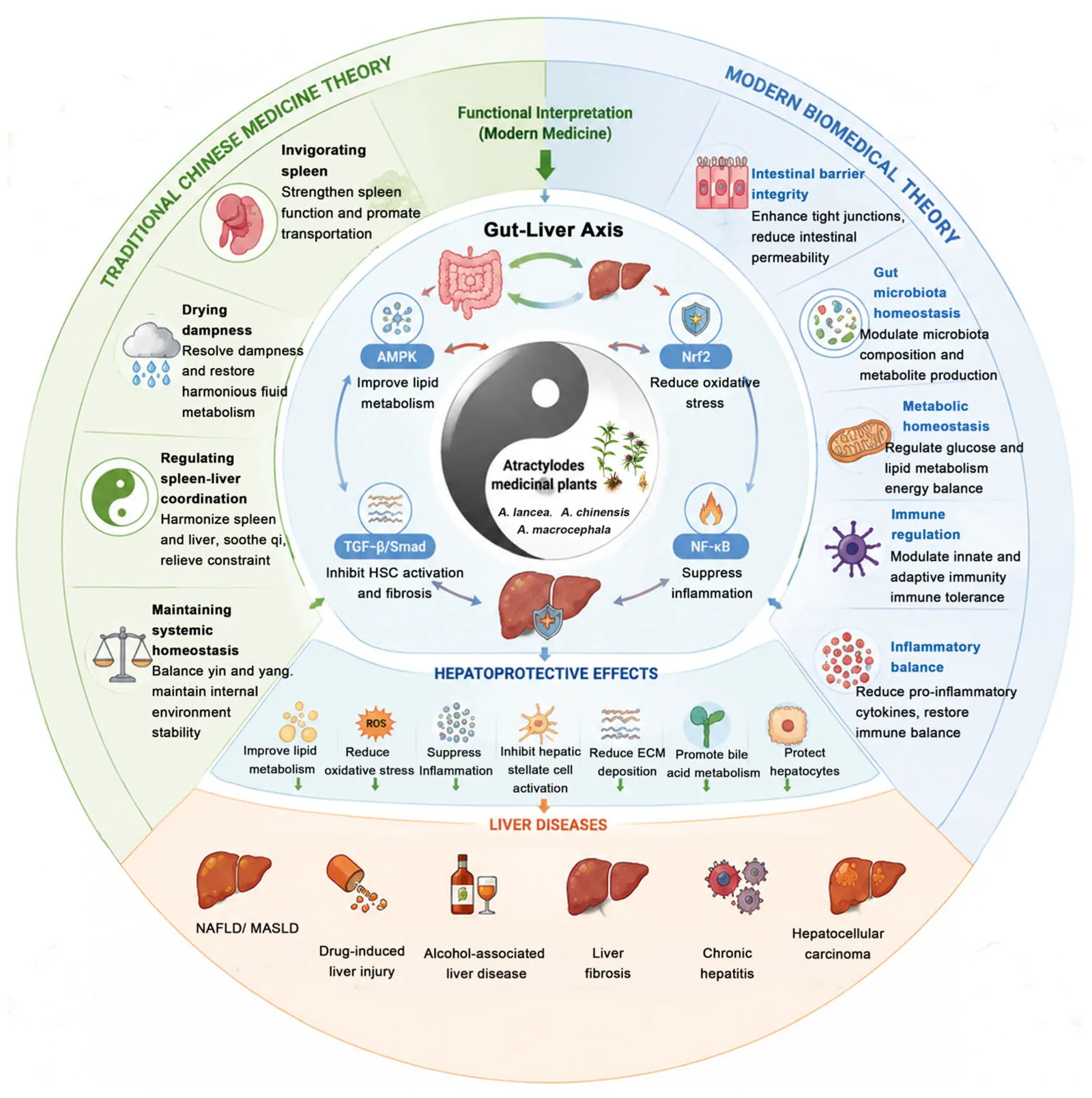
Traditional uses of *Atractylodes* and possible relationships with liver-related biological processes reported in experimental studies. Note: References [6,10,11,12,36,37,38,39,40,41,42,43,44,45,46]. Evidence base: The evidence summarized in this figure is derived from in vitro studies and in vivo animal models. Abbreviations: AMPK, AMP-activated protein kinase; Nrf2, nuclear factor erythroid 2-related factor 2; NF-κB, nuclear factor kappa B; TGF-β, transforming growth factor beta; NAFLD, non-alcoholic fatty liver disease; MASLD, metabolic dysfunction-associated steatotic liver disease. The arrows in this figure indicate proposed relationships or signaling-related changes rather than established causal mechanisms.

Modern pharmacological studies further indicate that the biological effects underlying the traditional function of “invigorating the spleen and drying dampness” may involve the synergistic actions of multiple bioactive constituents, including volatile oils, sesquiterpenes, and polysaccharides [41]. Representative compounds include atractylon, atractylodin, and atractylenolides [42]. These constituents not only regulate gastrointestinal motility, improve intestinal barrier integrity, and modulate gut microbiota composition, but may also participate in the regulation of liver diseases through the gut–liver axis [43,44]. Furthermore, several molecular pathways have been reported to be modulated by *Atractylodes*-derived compounds in experimental liver disease models. In metabolic liver diseases, particularly metabolic dysfunction-associated steatotic liver disease (MASLD), *Atractylodes*-derived compounds have been associated with regulation of AMPK-related signaling pathways, accompanied by changes in hepatocellular energy metabolism and reduced lipid accumulation in experimental settings [11,45]. In the context of oxidative stress during hepatic injury, these compounds have been linked to modulation of the Nrf2-mediated antioxidant defense system and altered expression of antioxidant enzymes, correlating with reduced markers of oxidative damage [9]. Meanwhile, *Atractylodes*-derived bioactive constituents have been reported to suppress NF-κB activation and pro-inflammatory cytokine release in experimental models, with associated attenuation of inflammatory responses [12]. In models of hepatic fibrosis, *Atractylodes*-derived compounds have been shown to affect the TGF-β/Smad signaling pathway, with reported changes in hepatic stellate cell activation and extracellular matrix deposition associated with reduced fibrotic progression [12,46]. Collectively, these experimental observations identify several liver-related biological processes that may provide a possible biomedical perspective for interpreting certain traditional uses of *Atractylodes* (Figure 1). However, these associations should not be regarded as demonstrating biological equivalence between traditional Chinese medicine concepts and specific molecular pathways.

## 5. Preclinical Pharmacological Effects and Proposed Mechanisms of *Atractylodes* in MASLD

Metabolic dysfunction-associated steatotic liver disease (MASLD) is a chronic, progressive liver disease characterized by hepatic steatosis and cardiometabolic abnormalities, driven by a combination of metabolic disorders—such as insulin resistance and overnutrition—and genetic susceptibility [47,48]. The 2023 global multi-society consensus replaced the previous term “non-alcoholic fatty liver disease” (NAFLD) with MASLD. Non-alcoholic steatohepatitis (NASH) was correspondingly renamed metabolic dysfunction-associated steatohepatitis (MASH) [49,50]. The 2024 EASL-EASD-EASO Joint Guidelines define MASLD as the presence of hepatic steatosis and at least one cardiometabolic risk factor, in the absence of alcohol exposure that meets the exclusion criteria; its disease spectrum can progress from simple steatosis to MASH, fibrosis, cirrhosis, and even hepatocellular carcinoma [51]. At the pathological level, the core mechanism begins with an imbalance in hepatic lipid metabolism. An increase in free fatty acids derived from peripheral lipolysis, combined with abnormally active de novo lipid synthesis in the liver and a reduction in lipid export in the form of very-low-density lipoproteins, leads to abnormal accumulation of triglycerides within hepatocytes. Microscopically, this manifests as macrovesicular steatosis, resulting in simple fatty liver [52]. Subsequent progressive damage is mediated by multiple factors: the accumulation of lipotoxic intermediates (such as free cholesterol and saturated fatty acids) triggers endoplasmic reticulum stress and oxidative stress, which not only directly damage organelles but also are associated with the induction of novel forms of cell death—such as pyroptosis and ferroptosis—involving pathways including the NLRP3 inflammasome. Factors released by damaged hepatocytes, together with endotoxins derived from gut dysbiosis, jointly activate hepatic immune cells, triggering persistent inflammation characterized by ballooning of hepatocytes; meanwhile, in an attempt to repair the damage, hepatic stellate cells are abnormally activated, leading to excessive extracellular matrix deposition originating in the central venous zone, which results in liver fibrosis and ultimately drives the progression of the disease toward cirrhosis and hepatocellular carcinoma [53,54]. This process does not evolve linearly but rather reflects network-based regulatory mechanisms involving metabolic disorders, genetic background, the gut microbiome, and cellular stress responses, which constitute the core challenges in current clinical diagnosis and new drug development.

### 5.1. Lipid Metabolic Reprogramming and Regulation of Fat Accumulation

Research on the active ingredients of AR and AMR in lipid metabolism and fat accumulation has involved both individual pathways and potential multi-target mechanisms in preclinical models. Current research indicates that AR water extracts have been associated with increased SIRT1 and AMPK expression in white adipose tissue of high-fat diet-induced obese mice, accompanied by changes in adipogenesis-related and fatty acid oxidation-related gene expression, and correlated with reduced weight gain and adipose tissue expansion [55]. This observation suggests that the SIRT1/AMPK pathway may contribute to the effects of AR in experimental obesity models. Further studies on AR-active monomers have provided additional mechanistic insights. Atractylodin has been reported to bind to AMPK in silico; in cellular models, it has been associated with modulation of SREBP-1c-regulated lipogenic genes (Srebf1, Fasn, Scd2, Dgat2) and PPARα-regulated fatty acid oxidation genes, accompanied by changes consistent with a shift from lipid synthesis toward catabolism [11,56,57]. Such structurally well-defined phytochemicals provide strong support for elucidating the chemical basis and mechanisms of action of AR-active compounds.

In addition, studies on AR polysaccharides have provided evidence for additional mechanisms relevant to lipid metabolism. In methionine- and choline-deficient models, AR polysaccharides have been associated with reduced hepatic steatosis, decreased hepatic lipid content, and lower triglyceride levels, suggesting effects on the hepatic lipid profile in this experimental setting [58]. Polysaccharide components are typically difficult to absorb directly into hepatocytes; their effects may therefore be partly mediated through modulation of the gut microbiome and its metabolites. This proposed mechanism is consistent with the growing body of evidence implicating the gut–liver axis in metabolic liver diseases [59]. In addition, several novel compounds, including phenolic glycosides, furanolignin glycosides, and pyrazine derivatives, have been reported to exhibit hepatoprotective activity under experimental conditions [60]. This further expands the range of potential bioactive components associated with metabolic liver injury.

### 5.2. Activation of the Oxidative Stress Defense System

Oxidative stress is considered a key driver in the onset and progression of various liver diseases, including MASLD, while several active components in AR and AMR have been associated with potential hepatoprotective effects in experimental models. Among the pathways implicated, Nrf2-related signaling has been frequently reported in experimental studies and represents a proposed mechanism associated with the antioxidant effects of *Atractylodes*-derived compounds. Nrf2 regulates the expression of antioxidant and ferroptosis-related proteins, including GPX4, FTH1, and SLC7A11. In experimental MASLD models, atractylodin treatment was associated with increased Nrf2 nuclear translocation and expression of these proteins, together with reduced iron, ROS, and MDA levels and increased GSH levels [9,61]. These changes were accompanied by reduced oxidative-stress- and ferroptosis-related indices, suggesting that Nrf2-related signaling may contribute to the observed effects. In addition to Nrf2 pathway modulation, other AMR components have been reported to affect antioxidant responses in experimental models. For example, a study reported that, in a rat model, atractylon can alleviate oxidative damage to the liver and DNA damage, an effect that may be related to its free radical scavenging activity [62], while AMR polysaccharides significantly increase the activity of superoxide dismutase (SOD), GSH, and glutathione peroxidase (GSH-Px), reduce MDA and ROS levels, and improve liver tissue histopathology and serum transaminase levels [63]. It is worth noting that purified AMR polysaccharide fractions have been associated with greater reductions in MDA, greater increases in antioxidant enzyme activity, and greater alleviation of inflammatory infiltration compared with crude extracts; moreover, these effects were accompanied by changes in Nrf2-related signaling, suggesting possible involvement of this pathway. Furthermore, AR extracts from various sources have demonstrated free radical scavenging activity in vitro and have shown no cytotoxicity at the concentrations tested, providing preliminary experimental support for the potential application of AR in oxidative stress research [64]. Collectively, these experimental observations indicate that *Atractylodes*-derived compounds are associated with changes in oxidative stress-related markers and antioxidant pathways in preclinical models.

### 5.3. Regulation of the Inflammatory Microenvironment

Imbalances in the inflammatory microenvironment are considered key drivers in the progression of liver disease from steatosis to hepatitis, fibrosis, and ultimately cancer. In preclinical models, active components in AMR, particularly AMR polysaccharides, have been associated with modulation of inflammatory responses and immune homeostasis at multiple levels, with effects observed on several signaling nodes. Studies have reported that AMR treatment is associated with reduced TLR4/MyD88/NF-κB-related signaling and lower levels of pro-inflammatory cytokines, including TNF-α, IL-1β, and IL-6, together with increased levels of anti-inflammatory mediators such as IL-10 or IL-4 in experimental models [12,65,66]. Mechanistically, TLR4 serves as a key receptor for recognizing pathogen- and injury-associated molecular patterns; its activation recruits MyD88 and initiates nuclear translocation of NF-κB, thereby inducing expression of downstream inflammatory genes.

**Figure 2:**
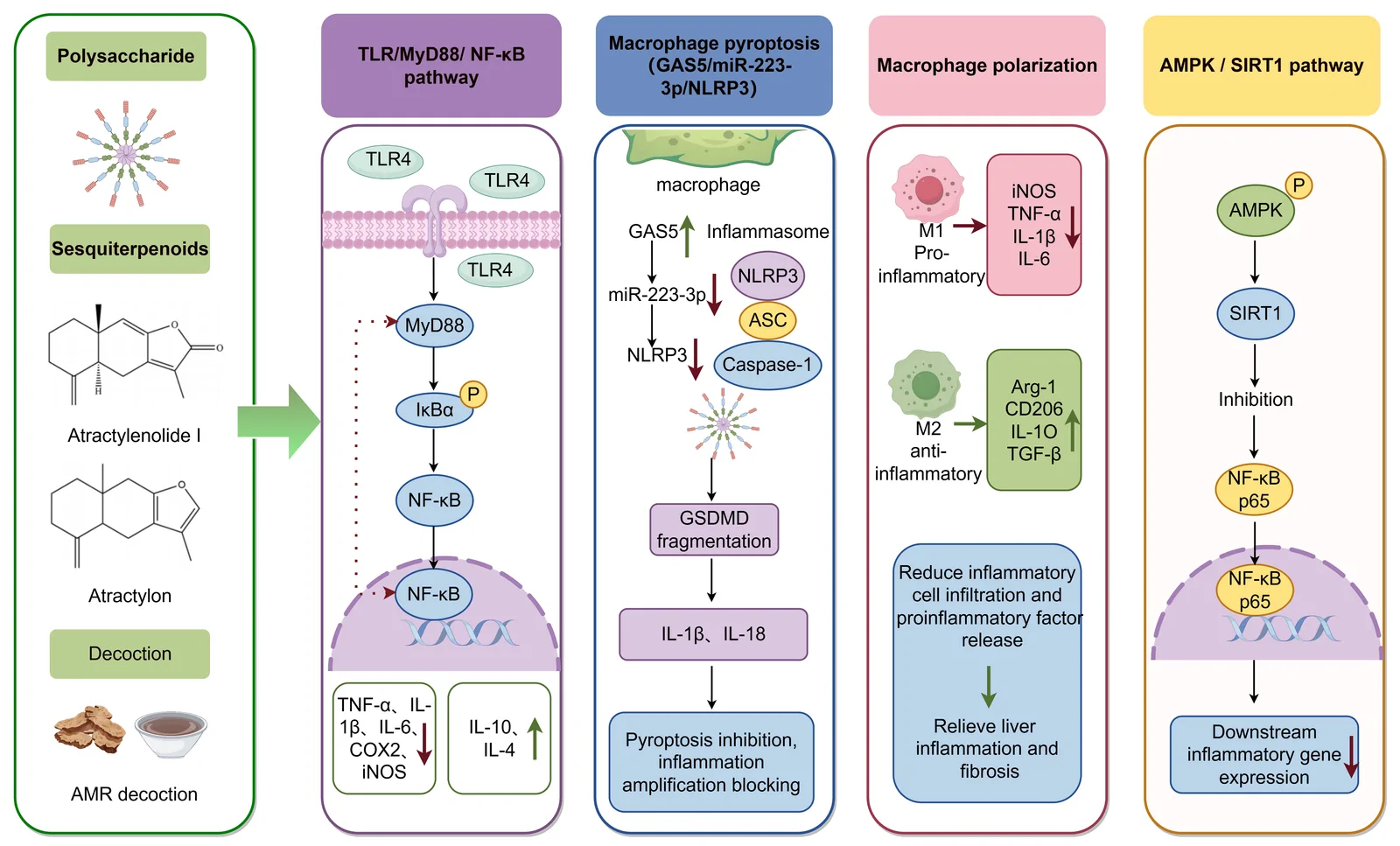
Preclinical evidence for modulation of inflammatory pathways by *Atractylodes*-derived preparations in MASLD. Note: References [12,58,65,66,67,68,69]. Evidence base: The evidence summarized in this figure is derived from in vitro studies and in vivo animal models. Abbreviations: AMPK, AMP-activated protein kinase; GAS5, growth arrest-specific transcript 5; iNOS, inducible nitric oxide synthase; IL, interleukin; M1, classically activated macrophage; M2, alternatively activated macrophage; miR-223-3p, microRNA-223-3p; MyD88, myeloid differentiation primary response 88; NF-κB, nuclear factor kappa B; NLRP3, NOD-like receptor family pyrin domain-containing 3; SIRT1, sirtuin 1; TLR4, Toll-like receptor 4; TNF-α, tumor necrosis factor alpha. The arrows in this figure indicate proposed relationships or signaling-related changes rather than established causal mechanisms.

In addition to the observed suppression of inflammatory signaling pathways, AMR has also been reported to play a role in regulating immune cell function and influencing the liver’s immune microenvironment in preclinical settings. More in-depth mechanistic studies have reported that AMR polysaccharides are associated with reduced NLRP3 inflammasome activation in macrophages. Specifically, treatment with AMR polysaccharides has been observed to correlate with modulation of the competitive endogenous RNA (ceRNA) relationship between the long non-coding RNA GAS5 and miR-223-3p, which is accompanied by decreased macrophage pyroptosis and attenuated sustained amplification of inflammation [67]. Similarly, AR polysaccharide treatment has also been associated with changes in macrophage polarization, including reduced M1-related and increased M2-related markers, accompanied by lower inflammatory-cell infiltration and pro-inflammatory mediator levels [58,68]. These observations suggest that modulation of macrophage phenotype may contribute to the inflammatory changes observed in these experimental models. In addition, AMR decoction treatment was associated with increased AMPK/SIRT1-related signaling and reduced NF-κB-related signaling, accompanied by changes in immune-related markers in experimental models [69]. In summary, these experimental observations suggest that the active ingredients of AR and AMR may influence inflammatory responses through multiple pathways, including TLR4/NF-κB signaling, macrophage pyroptosis, and polarization modulation, potentially involving a multi-level regulatory network (Figure 2).

### 5.4. Reported Effects on Gut–Liver Axis-Related Parameters and Gut Microbiota

*Atractylodes*-derived preparations have been associated with changes in gut microbiota composition, intestinal barrier-related markers, circulating endotoxin-related indices, and hepatic inflammatory and metabolic parameters in experimental models. Studies of AR polysaccharides and related preparations reported increased expression of intestinal tight-junction proteins, including occludin and claudin-1, accompanied by reduced circulating LPS-related indices [70,71]. These changes were also accompanied by reduced hepatic TLR4/MyD88/NF-κB-related signaling and inflammatory cytokine levels. Collectively, these observations suggest that intestinal barrier-related changes and reduced endotoxin exposure may contribute to some of the hepatic effects observed in these models.

In terms of gut microbiota, AMR and AR polysaccharides have been associated with changes in microbial composition in experimental models. Reported changes include reductions in the Firmicutes/Bacteroidetes ratio and in the relative abundance of taxa such as *Faecalibaculum rodentium*, *Lachnospiraceae*, and *Blautia*, together with increased abundance of *Muribaculaceae*, *Lactobacillus*, and *Bifidobacterium* [72,73]. These microbial changes were accompanied by alterations in intestinal barrier-related, metabolic, and immune-related parameters, including changes in carbohydrate-metabolizing enzymes and microbiota-derived metabolites such as short-chain fatty acids. However, the available evidence does not establish that the observed changes in microbiota composition directly mediate the improvements in intestinal or hepatic outcomes. Similarly, AMR extract treatment was associated with changes in gut microbiota composition, including increased abundance of *Lactobacillus*, *Bifidobacterium*, and *Akkermansia muciniphila*, together with changes in intestinal barrier-related indices, reduced LPS-related parameters, and modulation of HDL/LPS- and reverse-cholesterol-transport-related markers [74]. These changes were accompanied by reduced hepatic lipid accumulation and oxidative-stress-related indices.

Furthermore, the protective effects of AMR on the gut–liver axis extend to broader metabolic and neuroendocrine networks. For example, AMR polysaccharides not only improve liver pathology in MASH by regulating the gut microbiota and metabolites but also alleviate associated anxiety and depression-like behaviors, suggesting that the gut–liver–brain axis may be involved [72]. At the same time, AR and AMR polysaccharides have been associated with changes in autophagy-related signaling, AMPK/PPARα-related pathways, and p53/mTOR signaling in experimental models, accompanied by improvements in insulin resistance, lipid accumulation, and hepatocyte injury-related parameters [73,75]. Overall, experimental studies have reported changes in gut microbiota composition, intestinal tight-junction proteins, circulating LPS-related indices, and hepatic inflammatory and metabolic markers following treatment with *Atractylodes*-derived preparations. These findings suggest that gut–liver axis-related processes may contribute to some observed effects. However, the relative contributions of local intestinal actions, microbiota-derived metabolites, and systemic exposure remain uncertain, particularly for poorly absorbed polysaccharide fractions.

### 5.5. Autophagy-Related Changes Reported in Different Experimental Models

As a core mechanism for maintaining intracellular homeostasis, cellular autophagy plays a critical role in hepatic energy metabolism, lipid balance, and stress adaptation. Abnormal autophagy is closely associated with the onset and progression of various liver diseases, including metabolic dysfunction-associated steatohepatitis (MASH) and acute-on-chronic liver failure (ACLF). Active compounds in *Atractylodes*, including AR polysaccharides and atractylenolide I, have been associated with modulation of autophagy signaling pathways and with changes in cellular metabolic networks in experimental models. In a methionine-choline-deficient (MCD) diet-induced MASH model, AR polysaccharides have been reported to be associated with reduced lipid accumulation, inflammatory markers, and hepatocyte injury, accompanied by inhibition of p53/mTOR signaling and decreased autophagic flux and ferroptosis levels [58,76]. This finding suggests that, under certain pathological conditions, excessive autophagy may exacerbate cellular damage, whereas moderate inhibition of autophagy may be associated with improved metabolic parameters under specific experimental conditions. In contrast, in an ACLF model, atractylenolide I has been associated with autophagy-promoting effects: it has been reported to inhibit mTOR activity, enhance autophagic flux, preserve mitochondrial integrity, and reduce hepatocyte apoptosis, accompanied by changes in systemic inflammatory markers and liver function parameters [10,77]. These studies report different directions of autophagy-related changes under distinct experimental conditions. AR polysaccharides were associated with reduced autophagy-related activity in a methionine-choline-deficient metabolic liver disease model, whereas atractylenolide I was associated with enhanced autophagic activity in an acute-on-chronic liver failure model. Because these observations were obtained using different compounds, disease models, and experimental conditions, they should not be interpreted as evidence that *Atractylodes*-derived compounds intrinsically exert adaptive or bidirectional regulation of autophagy. Rather, the available findings suggest that the relationship between individual *Atractylodes*-derived compounds and autophagy is context dependent and requires direct comparative investigation.

In addition, several studies have reported associations between AMR-mediated autophagy modulation and changes in metabolic parameters in preclinical models. For example, in a MASH model, an Alisma–Rhizoma decoction (containing 50% AMR) was reported to be associated with reduced autophagosome numbers and LC3-II expression, accompanied by changes in hepatic superoxide dismutase activity and malondialdehyde levels, suggesting a possible association between autophagy modulation and redox-related changes [78]. Furthermore, modulation of the p53/mTOR pathway by AR constituents has been associated with both autophagic changes and reduced ferroptosis markers, accompanied by alterations in lipid peroxidation-related parameters. In parallel, atractylenolide I-mediated autophagy has been associated with changes in mitochondrial function, energy metabolism markers, and ROS levels in experimental models. Therefore, the available studies indicate that *Atractylodes*-derived preparations and individual compounds are associated with changes in autophagy-related signaling and metabolic parameters in different experimental models.

In summary, despite these findings, the evidence concerning *Atractylodes*-derived interventions in MASLD remains predominantly preclinical and methodologically heterogeneous. The available studies employ different extracts, polysaccharide fractions, purified compounds, doses, and experimental models, which limits direct comparison across studies. In many cases, pathway involvement is inferred from changes in protein expression or phosphorylation rather than direct target engagement. Moreover, experimental doses and exposures have not been systematically linked to human pharmacokinetic data. The divergent autophagy-related findings obtained with different compounds and disease models further illustrate this uncertainty. Therefore, the current evidence supports biological and hepatoprotective activity in specific experimental models but does not establish therapeutic efficacy in patients with MASLD.

## 6. Preclinical Pharmacological Effects and Proposed Mechanisms of *Atractylodes* in ALD

Alcohol-associated liver disease (ALD) remains one of the leading causes of chronic liver disease worldwide, with severity ranging from simple steatosis to steatohepatitis, fibrosis, cirrhosis, and even hepatocellular carcinoma [79]. The American Association for the Study of Liver Diseases (AASLD) Clinical Practice Guidelines (2020) emphasize that ALD management should be based on the assessment of drinking behavior and alcohol use disorder (AUD), sustained abstinence, and the integration of psychosocial and addiction medicine interventions; however, effective medications for advanced ALD remain limited [80]. Its pathogenesis primarily involves ethanol- and acetaldehyde-induced metabolic disorders, CYP2E1-mediated reactive oxygen species (ROS) production, mitochondrial damage, and lipid peroxidation, which promote LPS translocation through disruption of the intestinal barrier, thereby activating TLR4-related innate immune and inflammatory responses. Therefore, metabolic dysregulation, oxidative stress, inflammation, and gut–liver axis dysfunction represent important pathological features of ALD.

### 6.1. Effects on Oxidative-Stress-Related Markers and Signaling

Available preclinical studies indicate that, in experimental models of alcohol-induced liver injury, AR and AMR extracts are associated with changes in various redox-related markers [81]. Studies have confirmed that, in a rat model of combined metabolic dysfunction and alcohol-induced liver injury established by a high-sugar, high-fat diet with excessive alcohol intake, AMR extract crystals (BZEP) and their self-microemulsion formulation (BZEPWR) significantly increased serum antioxidant markers, including GSH and SOD, while markedly reducing ROS levels [74]. In the same model, BZEP and BZEPWR also increased serum HDL-C and its subfractions, significantly reduced serum LPS levels, and downregulated the hepatic expression of inflammatory signaling proteins, including TLR4, MyD88, and NF-κB. This series of changes suggests that AMR extracts may be associated with attenuation of inflammatory markers, accompanied by improvements in intestinal barrier-related parameters and modulation of LPS/TLR4-mediated signaling, and correlated with changes in hepatic redox status. *Atractylodes* polysaccharides were similarly associated with increased antioxidant enzyme activities and reduced ROS and MDA levels, together with changes in Nrf2/HO-1 signaling [82]. In mice with alcohol-induced liver injury, methyl ferulic acid reduced CYP2E1 and NOX4-related indices and was accompanied by lower ROS and MDA levels and higher antioxidant enzyme activities [83]. These findings support an association between *Atractylodes*-derived interventions and modulation of oxidative-stress-related pathways in preclinical models. However, they do not by themselves establish restoration of redox homeostasis or direct molecular target engagement.

### 6.2. Inhibition of Alcohol-Induced Inflammatory Responses and Hepatocyte Protection

Alcohol-associated liver injury involves interacting processes including ethanol metabolism, oxidative stress, inflammatory signaling, and hepatocellular damage. In preclinical studies, AR- and AMR-derived constituents have been associated with changes in several of these processes, including alcohol-metabolism-related enzymes, oxidative-stress markers, and inflammatory signaling pathways. These observations suggest that multiple biological processes may contribute to the hepatoprotective effects reported in experimental models, although the causal relationships among these changes remain incompletely defined.

In experimental models of alcohol-induced liver injury, methyl ferulic acid (MFA) treatment was associated with reduced CYP2E1- and NOX4-related indices, lower ROS levels, increased antioxidant enzyme activities, reduced phosphorylation of MAPK-related proteins including JNK, p38, and ERK, and decreased expression of pro-inflammatory mediators such as TNF-α, IL-1β, and IL-6 [83,84]. These changes were accompanied by reduced hepatocellular injury, suggesting that oxidative-stress- and MAPK-related inflammatory pathways may contribute to the observed effects of MFA. In a carcinogen-initiated, alcohol-promoted precancerous lesion model, luteolin supplementation was associated with reduced hepatic inflammatory lesions and steatosis and a lower incidence of precancerous lesions, together with changes in SIRT1 activity, the acetylated/total FoxO1 ratio, and PGC-1α-related signaling [85]. These findings are consistent with possible involvement of SIRT1-related metabolic and inflammatory regulation; however, they do not establish that modulation of this pathway directly prevents progression from alcohol-associated inflammation to malignancy. In addition, an in vitro comparative study reported that ferulic acid was associated with reduced ethanol-induced changes in cell membrane permeability and inflammatory mediator levels [86].

Overall, the available preclinical studies report coordinated changes in alcohol-metabolism-related enzymes, oxidative-stress markers, MAPK- and Nrf2/HO-1-related signaling, inflammatory mediators, and SIRT1-associated metabolic responses following treatment with selected AR- and AMR-derived constituents. These findings suggest that multiple stress-, inflammatory-, and metabolic-related pathways may contribute to the observed hepatoprotective effects, but they do not establish a unified causal regulatory network. Further studies using direct pathway-intervention approaches and pharmacologically relevant exposure conditions are required to clarify the contribution of individual pathways to the effects observed in ALD models.

### 6.3. Improvement in Lipid Reprogramming and Accumulation Associated with Alcohol Metabolism Disorders

In states of alcohol metabolism dysfunction, the liver’s cholesterol homeostasis is severely disrupted, cholesterol synthesis becomes abnormally active, while the reverse transport clearance pathway is inhibited, leading to massive accumulation of cholesterol esters within hepatocytes. At the molecular level, LXR—a central regulator of cholesterol metabolism—has been implicated as one of the molecular nodes through which AMR-derived compounds may modulate cholesterol homeostasis. In experimental studies, AMR treatment has been associated with increased expression of proteins involved in reverse cholesterol transport (RCT), including apolipoprotein A1 (Apo-A1), lecithin-cholesterol acyltransferase (LCAT), and scavenger receptor B type I (SR-BI), together with changes in HDL-C-related indices [87,88]. AMR treatment has also been associated with reduced HMG-CoA reductase activity and changes in the balance between acyl-CoA cholesterol acyltransferase (ACAT) and LCAT. These findings suggest that cholesterol synthesis, esterification, and reverse transport may contribute to the observed changes in hepatic cholesterol accumulation, although the relative contribution of each process remains to be established.

In addition to these cholesterol-related changes, other AMR-derived constituents have been associated with alterations in lipid-metabolism-related pathways [89]. Methyl ferulic acid treatment was associated with reduced miR-378b expression and increased CaMKK2/AMPK-related signaling, together with decreased expression of lipogenesis-related factors, including SREBP-1c and FASN, and increased expression of PPARα, CPT1, and MTTP [90]. These molecular changes were accompanied by reduced triglyceride and cholesterol accumulation in hepatocytes. Collectively, these findings suggest that changes in lipid synthesis, fatty acid oxidation, lipid transport, and LXR-related cholesterol metabolism may contribute to the effects observed in experimental models; however, the specific mechanisms remain to be established.

### 6.4. Effects on Hepatocyte Survival and Apoptosis-Related Pathways

In the progression of alcohol-induced fatty liver disease, hepatocyte death is a central event associated with amplification of inflammatory responses and deterioration of liver function; therefore, reducing hepatocyte death represents an important therapeutic objective. Although reports specifically targeting alcohol models are limited, studies from other liver injury models may provide mechanistic hypotheses relevant to ALD. Studies of atractylenolide I in a model of acute-on-chronic liver failure (ACLF) have shown that treatment was associated with increased autophagy-related activity, changes in mTOR signaling, improved mitochondrial-function-related indices, and reductions in fibrosis-, inflammation-, and necrosis-related parameters. In vitro experiments further showed increased hepatocyte survival and reduced apoptosis, while the mTOR activator MHY1485 partially attenuated these effects, supporting the involvement of mTOR-related autophagy signaling in the observed response [10,91,92]. Studies on ferulic acid (FA) in acetaminophen-induced acute liver injury have similarly reported changes in AMPK- and autophagy-related signaling together with reduced apoptosis-related indices. FA was associated with preservation of mitochondrial membrane potential, a reduced Bax/Bcl-2 ratio, decreased caspase-3 activation, and increased AMPK phosphorylation and autophagy-related activity; attenuation of these effects by the AMPK inhibitor compound C supports a contribution of AMPK-related signaling in this experimental model [93,94].

Furthermore, studies in mice with alcoholic liver injury have shown that methyl ferulic acid (MFA) treatment was associated with a reduced Bax/Bcl-2 ratio, decreased activation of caspase-3 and caspase-9, and reduced phosphorylation of MAPK-related proteins, including JNK, p38, and ERK, together with lower levels of hepatocyte apoptosis [83,95]. These findings are consistent with possible involvement of mitochondrial apoptosis- and MAPK-related signaling but do not establish that MFA directly promotes hepatocyte regeneration or post-injury liver regeneration. Active flavonoid compounds in *Atractylodes* have also been associated with reduced apoptosis-related markers in alcohol-induced liver injury models. Among these, naringin was associated with reduced caspase-3 activation, suggesting that apoptosis-related pathways may contribute to the observed hepatoprotective effects [96].

Taking these findings together, some of the available mechanistic evidence is derived from ACLF or acetaminophen-induced liver injury rather than ALD itself. Although mitochondrial dysfunction, oxidative stress, and apoptosis-related processes are relevant across these models, this overlap does not establish that the same compound–pathway relationships operate in alcohol-associated liver disease. Therefore, mTOR/autophagy-, AMPK/autophagy-, and ROS/MAPK-related pathways represent candidate mechanisms that may contribute to hepatocyte survival-related effects observed with atractylenolide I and ferulic acid derivatives in experimental settings. Future studies should further investigate these pathways in models of alcohol-associated liver disease to clarify their potential relevance to ALD.

### 6.5. Intestinal Barrier- and Gut–Liver Axis-Related Changes in Experimental ALD Models

In the pathogenesis of ALD, impaired intestinal barrier function and gut microbiota dysbiosis are important features associated with intestinal and hepatic inflammatory responses. Alcohol and its metabolites are associated with disruption of intestinal epithelial tight junctions, increased intestinal permeability, and, in experimental models, elevated portal or circulating levels of the intestinal endotoxin lipopolysaccharide (LPS). These changes may contribute to activation of hepatic TLR4/MyD88/NF-κB-related inflammatory signaling and alterations in lipid metabolism. Within the gut–liver axis, intestinal immune signaling and microbiota-derived metabolites have been implicated in the interaction between intestinal dysfunction and hepatic injury. Studies have shown that *Lactobacillus rhamnosus* Gorbach-Goldin (LGG) treatment was associated with increased IL-22 expression, increased levels of the tight-junction proteins ZO-1 and claudin-1, improved intestinal barrier-related parameters, and reduced liver injury in experimental ALD models [97]. These findings support a possible contribution of intestinal immune signaling to the effects of microbial interventions but do not by themselves establish a complete causal pathway from IL-22 signaling to hepatic protection. Postbiotics derived from *Lactobacillus plantarum* have likewise been associated with changes in gut microbiota composition, including increased abundance of *Akkermansia muciniphila*, together with improvements in liver injury-related indices [98].

In addition to the microbial intervention strategies discussed above, bioactive components from *Atractylodes* species have been associated with changes in multiple gut–liver axis-related parameters in preclinical studies. In rats treated with crystallized AMR extracts or their microemulsion formulations, changes in gut microbiota composition were accompanied by increased ABCA1/LXR-related indices and HDL-C levels, reduced LPS-related parameters, and lower hepatic TLR4/NF-κB-related signaling [74]. These observations suggest that microbiota composition, cholesterol transport, endotoxin-related signaling, and hepatic inflammation may be jointly involved in the observed effects; however, the available evidence does not establish a sequential causal cascade among these processes. At the same time, AR polysaccharide treatment was associated with increased expression of intestinal tight-junction proteins, including ZO-1 and occludin, together with reduced serum levels of LPS, D-lactic acid, and diamine oxidase, consistent with improvements in intestinal barrier-related parameters. Treatment was also associated with changes in gut microbiota composition at multiple taxonomic levels, including a reduced Firmicutes/Bacteroidetes ratio, increased abundance of *Lactobacillus* and *Limosilactobacillus*, and increased relative abundance of species such as *Lactobacillus taiwanensis*, *Limosilactobacillus reuteri*, and *Akkermansia muciniphila*, together with reduced abundance of Tylosporaceae-related taxa [82]. However, these microbiota and barrier-related changes should not be interpreted as demonstrating that microbiota remodeling or reduced intestinal permeability directly mediates the hepatic effects. Overall, probiotic/postbiotic interventions and *Atractylodes*-derived preparations produce partially overlapping changes in intestinal barrier-related, microbiota, endotoxin-related, and hepatic inflammatory parameters in preclinical ALD models. These observations support further investigation of the gut–liver axis as a possible contributor to the observed hepatoprotective effects. The available evidence therefore provides a preclinical rationale for investigating gut-targeted approaches in ALD, while the causal relationships among microbiota changes, barrier function, endotoxin exposure, and hepatic outcomes remain to be clarified.

**Figure 3:**
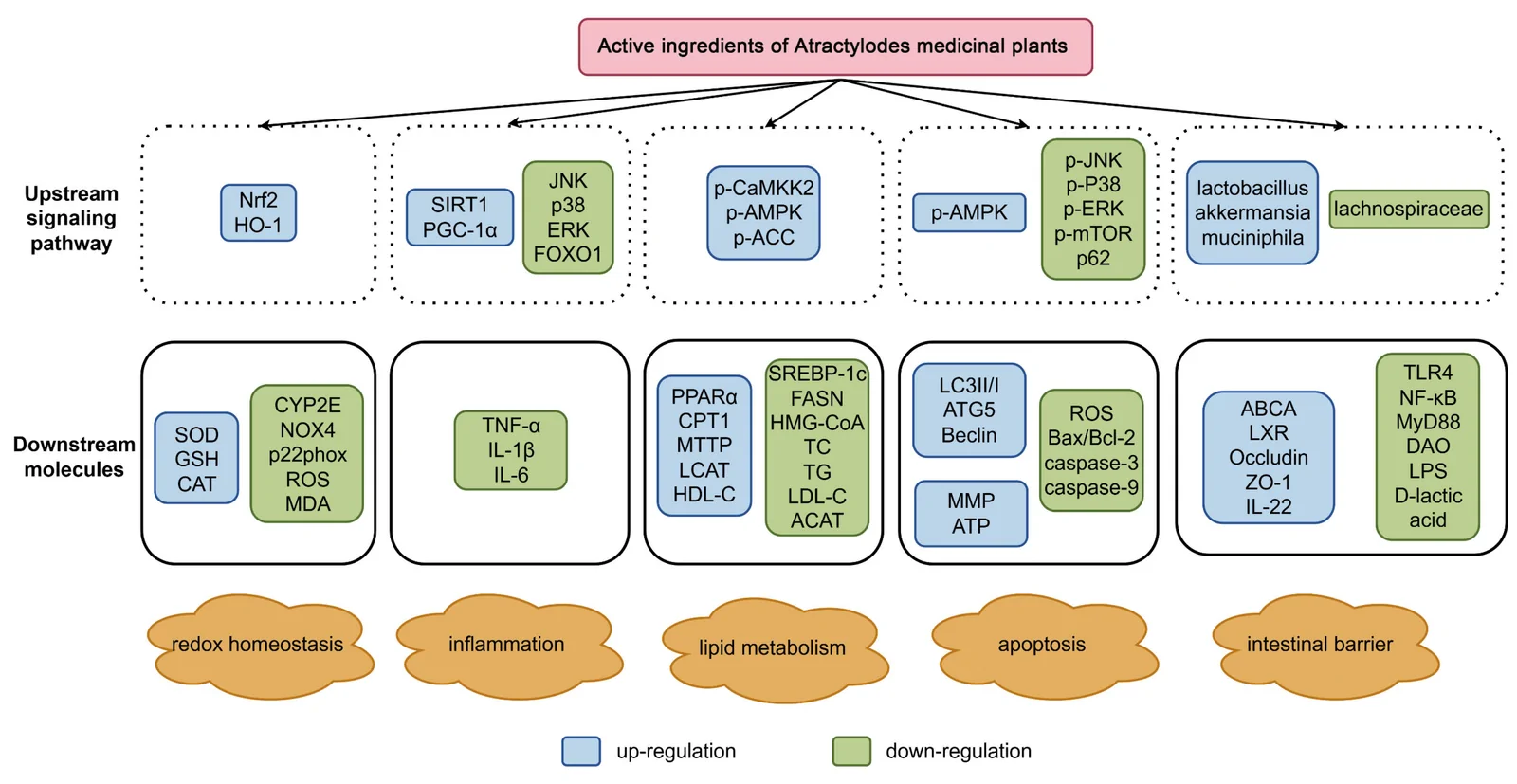
Reported pathways associated with *Atractylodes*-derived interventions in preclinical models of ALD. Note: References [74,81,82,83,84,85,86,87,88,89,90,97,98]. Evidence base: The evidence summarized in this figure is derived from in vitro studies and in vivo animal models. Abbreviations: ACC, acetyl-CoA carboxylase; ACAT, acyl-CoA cholesterol acyltransferase; AMPK, AMP-activated protein kinase; ATG5, autophagy-related 5; ATP, adenosine triphosphate; Bax, Bcl-2-associated X protein; Bcl-2, B-cell lymphoma 2; CaMKK2, calcium/calmodulin-dependent protein kinase kinase 2; CAT, catalase; CPT1, carnitine palmitoyltransferase 1; DAO, diamine oxidase; ERK, extracellular signal-regulated kinase; FASN, fatty acid synthase; FOXO1, forkhead box O1; GSH, glutathione; HDL-C, high-density lipoprotein cholesterol; HMG-CoA, 3-hydroxy-3-methylglutaryl coenzyme A; HO-1, heme oxygenase 1; IL, interleukin; JNK, c-Jun N-terminal kinase; LC3, microtubule-associated protein 1 light chain 3; LCAT, lecithin-cholesterol acyltransferase; LDL-C, low-density lipoprotein cholesterol; LPS, lipopolysaccharide; LXR, liver X receptor; MDA, malondialdehyde; MMP, mitochondrial membrane potential; mTOR, mechanistic target of rapamycin; MTTP, microsomal triglyceride transfer protein; MyD88, myeloid differentiation primary response 88; NF-κB, nuclear factor kappa B; NOX4, NADPH oxidase 4; Nrf2, nuclear factor erythroid 2-related factor 2; p38, p38 mitogen-activated protein kinase; PGC-1α, peroxisome proliferator-activated receptor gamma coactivator 1-alpha; PPARα, peroxisome proliferator-activated receptor alpha; ROS, reactive oxygen species; SIRT1, sirtuin 1; SOD, superoxide dismutase; SREBP-1c, sterol regulatory element-binding protein 1c; TC, total cholesterol; TG, triglyceride; TLR4, Toll-like receptor 4; TNF-α, tumor necrosis factor alpha; ZO-1, zonula occludens 1.

In summary, *Atractylodes*-derived interventions have been associated with changes in oxidative-stress-related markers, inflammatory signaling, lipid metabolism, intestinal barrier-related indices, and gut microbiota composition in preclinical models relevant to ALD (Figure 3). These findings support further investigation of several potentially involved biological processes. However, the available evidence is predominantly preclinical and does not establish that these pathways constitute a unified mechanism of action or that the observed effects translate into therapeutic efficacy in patients with ALD.

## 7. Preclinical Evidence for *Atractylodes*-Derived Compounds in Hepatic Fibrosis

**Figure 4:**
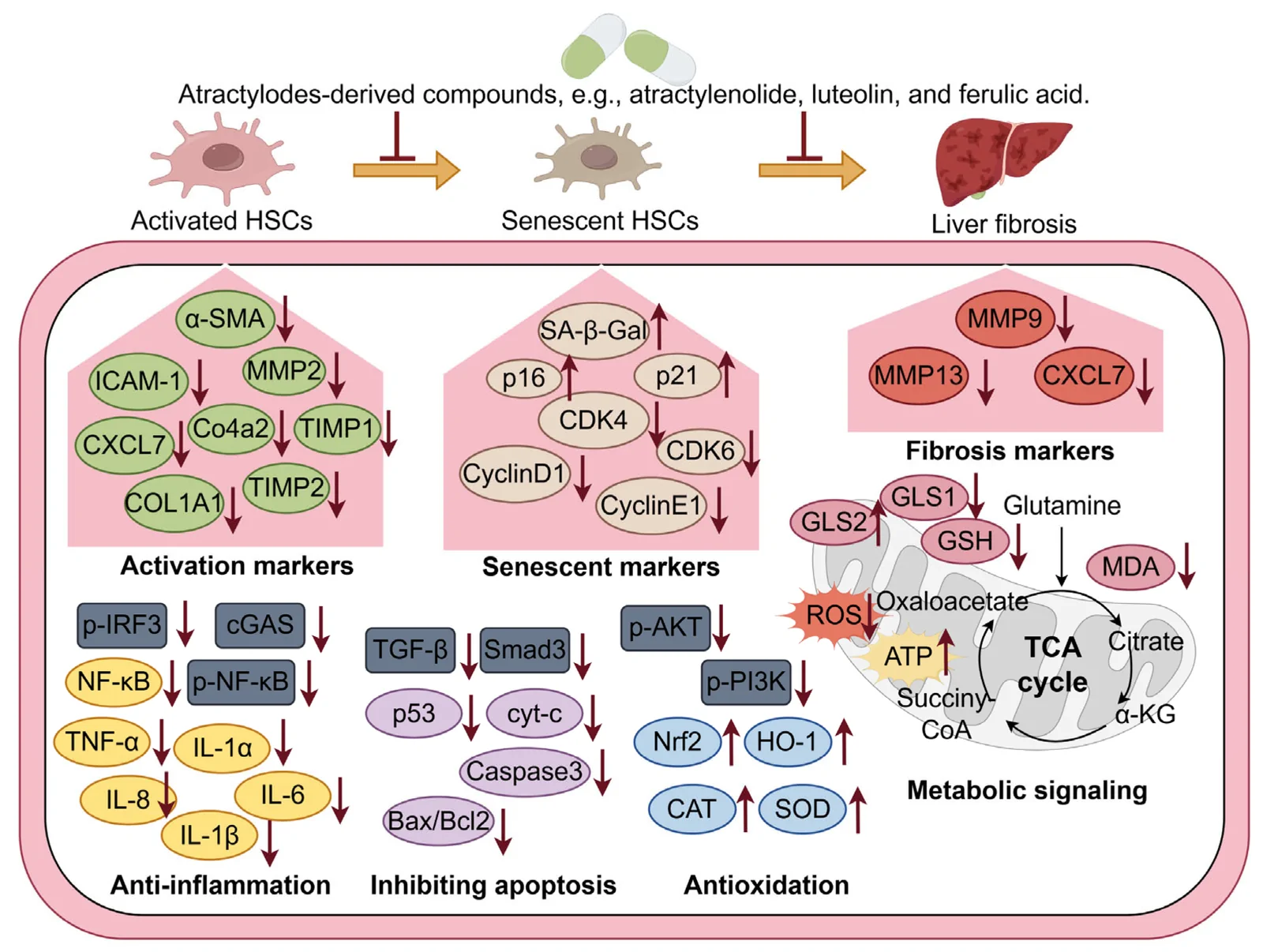
Preclinical evidence for effects of *Atractylodes*-derived compounds on hepatic stellate cell activation and fibrosis-related pathways. Note: References [99,100,101,102,103,104,105]. Evidence base: The evidence summarized in this figure is derived from in vitro studies and in vivo animal models. Abbreviations: α-KG, alpha-ketoglutarate; α-SMA, alpha-smooth muscle actin; AKT, protein kinase B; ATP, adenosine triphosphate; Bax, Bcl-2-associated X protein; Bcl-2, B-cell lymphoma 2; CAT, catalase; CDK, cyclin-dependent kinase; cGAS, cyclic GMP–AMP synthase; COL1A1, collagen type I alpha 1 chain; CoA, coenzyme A; CXCL7, C-X-C motif chemokine ligand 7; cyt-c, cytochrome c; GLS, glutaminase; GSH, glutathione; HO-1, heme oxygenase-1; HSCs, hepatic stellate cells; ICAM-1, intercellular adhesion molecule 1; IL, interleukin; IRF3, interferon regulatory factor 3; MDA, malondialdehyde; MMP, matrix metalloproteinase; NF-κB, nuclear factor kappa B; Nrf2, nuclear factor erythroid 2-related factor 2; p-AKT, phosphorylated AKT; p-IRF3, phosphorylated IRF3; PI3K, phosphoinositide 3-kinase; ROS, reactive oxygen species; SA-β-Gal, senescence-associated beta-galactosidase; Smad3, SMAD family member 3; SOD, superoxide dismutase; TCA, tricarboxylic acid; TGF-β, transforming growth factor beta; TIMP, tissue inhibitor of metalloproteinase; TNF-α, tumor necrosis factor alpha. The arrows in this figure indicate proposed relationships or signaling-related changes rather than established causal mechanisms.

The mechanisms by which *Atractylodes* medicinal materials may influence fibrosis-related processes involve multiple biological events, including changes in hepatic stellate cell (HSC) activation, extracellular matrix (ECM) turnover, oxidative-stress-related responses, and inflammatory signaling (Figure 4). Representative active compounds in *Atractylodes* medicinal materials have been associated with reduced TGF-β1 expression and decreased collagen-related indices in experimental fibrosis models [99,100]. In addition, luteolin treatment was accompanied by reduced TGF-β1/Smad-related signaling [101]. TGF-β1 is a well-recognized regulator of HSC transdifferentiation into myofibroblast-like cells and is closely associated with increased synthesis of type I and type III collagen. These findings suggest that TGF-β1/Smad-related signaling may contribute to the observed changes in HSC activation and collagen deposition. At the same time, atractylenolide III treatment has been associated with changes in ECM-related parameters, including reduced α-SMA and collagen I expression together with alterations in MMP/TIMP-related indices [102]. These observations are consistent with possible effects on both ECM production and turnover, although the available evidence does not establish that atractylenolide III directly enhances the degradation of previously deposited ECM. Collectively, these findings suggest that *Atractylodes*-derived compounds may be associated with changes in both collagen synthesis-related and ECM turnover-related processes in experimental fibrosis models. In addition, active components of AR and AMR have been associated with changes in oxidative-stress- and inflammation-related markers together with reduced HSC activation and fibrotic indices. Oxidative stress and inflammatory signaling are important contributors to persistent HSC activation, and small-molecule constituents such as atractylenolide III and ferulic acid have been reported to alter these parameters in experimental models [103,104,105]. These observations suggest that oxidative-stress- and inflammation-related pathways may contribute to the fibrosis-related effects observed with selected *Atractylodes*-derived compounds. However, they do not establish that these interventions disrupt a defined “injury–inflammation–fibrosis” causal cycle or act through a unified upstream antifibrotic mechanism.

It should be noted that evidence regarding antifibrotic activity also stems primarily from preclinical studies. Most studies have evaluated hepatic stellate cell activation, collagen deposition, α-SMA expression, or TGF-β/Smad-related signaling in cellular or animal models; however, the direct molecular targets and the interactions between the compounds and these targets have not yet been fully elucidated. Furthermore, differences in compounds, extract components, dosing regimens, and fibrosis models further limit the comparability among different studies. Therefore, while existing findings support the investigation of *Atractylodes*-derived interventions in fibrosis-related experimental systems, their clinical efficacy in preventing fibrosis or improving cirrhosis has not yet been established.

## 8. Preclinical Antitumor Activities of *Atractylodes*-Derived Bioactive Compounds in Hepatocellular Carcinoma

The malignant progression of hepatocellular carcinoma (HCC) involves multiple biological processes, including sustained proliferation, resistance to cell death, migration, and invasion. Preclinical studies have reported that selected AR- and AMR-derived compounds are associated with reduced viability and migration of HCC cells, together with changes in apoptosis- and invasion-related markers. In terms of apoptosis-related effects, atractylon treatment reduced mitochondrial membrane potential (ΔΨm), increased ROS levels, decreased Bcl-2 expression, increased Bax expression, and increased cleaved caspase-3 in HepG2, SMMC-7721, and MHCC97H cells, together with reduced cell viability [14,106]. In terms of migration- and invasion-related effects, atractylon treatment was associated with reduced EMT-related features and decreased expression of MMP-2 and MMP-9, accompanied by reduced migration and invasion in experimental assays [14]. In vitro migration assays and preclinical tumor models provide evidence of anti-migratory and antitumor activity [107]; however, these findings should not be interpreted as demonstrating clinically established anti-metastatic efficacy.

**Figure 5:**
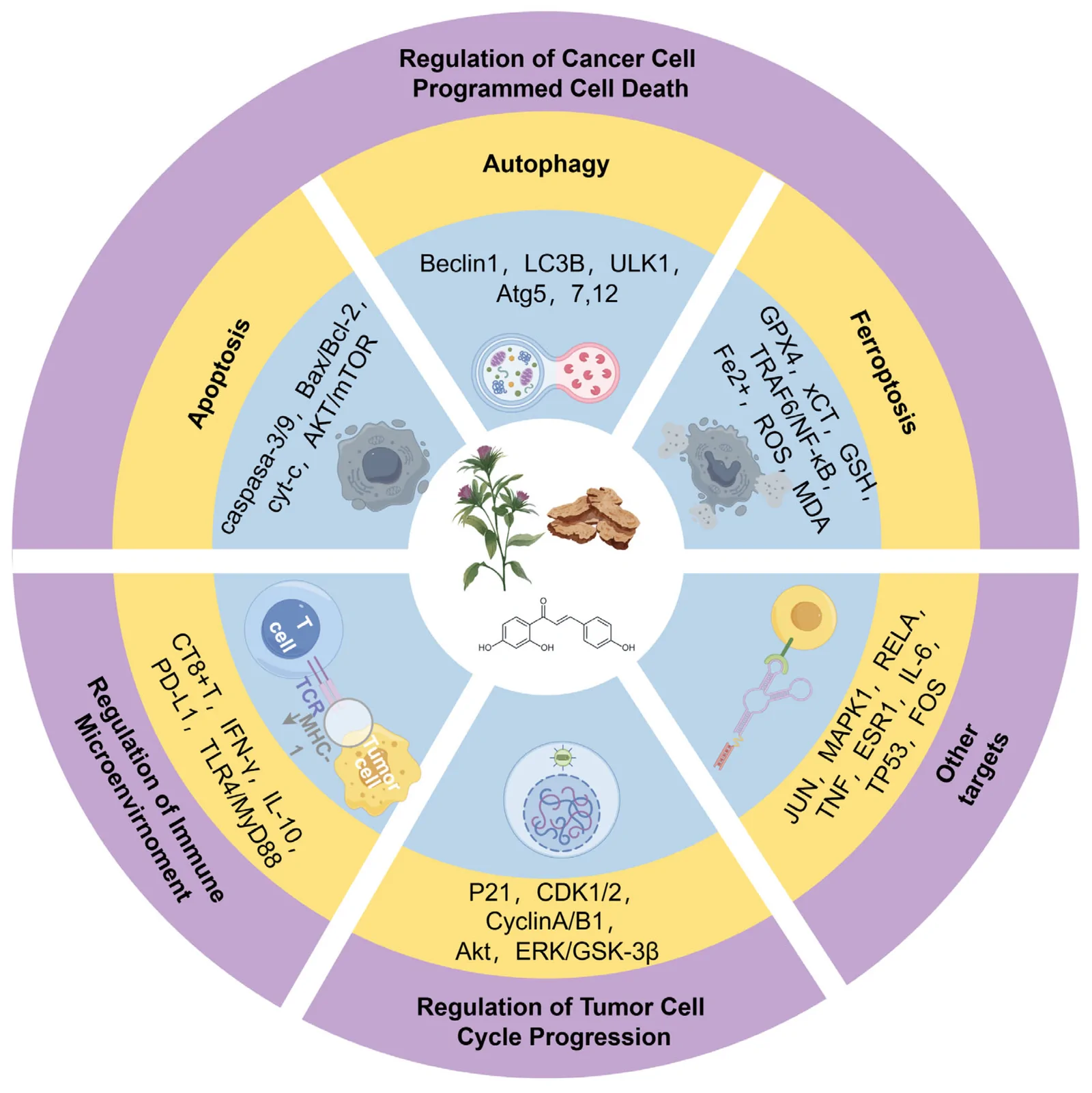
Preclinical antitumor activities of *Atractylodes*-derived compounds in hepatocellular carcinoma. Note: References [14,106,107,108,109,110,111,112]. Evidence base: The figure integrates computational predictions with preclinical experimental evidence derived from in vitro studies. Abbreviations: AKT, protein kinase B; ATG, autophagy-related protein; Bax, Bcl-2-associated X protein; Bcl-2, B-cell lymphoma 2; CD8, cluster of differentiation 8; CDK, cyclin-dependent kinase; cyt-c, cytochrome c; ERK, extracellular signal-regulated kinase; ESR1, estrogen receptor 1; FOS, Fos proto-oncogene, AP-1 transcription factor subunit; GPX4, glutathione peroxidase 4; GSH, glutathione; GSK-3β, glycogen synthase kinase 3 beta; IFN-γ, interferon gamma; IL, interleukin; JUN, Jun proto-oncogene, AP-1 transcription factor subunit; LC3B, microtubule-associated protein 1 light chain 3 beta; MAPK1, mitogen-activated protein kinase 1; MDA, malondialdehyde; MHC, major histocompatibility complex; mTOR, mechanistic target of rapamycin; MyD88, myeloid differentiation primary response 88; NF-κB, nuclear factor kappa B; PD-L1, programmed death-ligand 1; ROS, reactive oxygen species; TCR, T-cell receptor; TLR4, Toll-like receptor 4; TNF, tumor necrosis factor; TP53, tumor protein p53; TRAF6, TNF receptor-associated factor 6; ULK1, Unc-51-like autophagy activating kinase 1; xCT, cystine/glutamate antiporter (SLC7A11).

Network pharmacology analyses have predicted associations between *Atractylodes*-derived constituents and proteins including JUN, MAPK1, RELA, TNF, and TP53, providing hypotheses for subsequent experimental investigation [108]. Separate experimental studies have reported that atractylon treatment is associated with changes in PI3K/AKT/mTOR-related signaling and autophagy- and apoptosis-related markers in HCC cells [109]. Atractylenolide II has also been associated with changes in proliferation, ferroptosis-related markers, and immune-related signaling, including xCT/GPX4-, ROS/Fe^2+^-, and TRAF6/NF-κB-related indices [110]. These observations suggest that several cell-death-, redox-, and immune-related processes may be involved. Meanwhile, novel sesquiterpene dimers isolated from AMR, including atramacronins A–P, have shown cytotoxic activity against HCC cell lines including Hep3B, HepG2, and Huh7 in vitro [111]. Studies of atractylenolides I–III have additionally reported changes in angiogenesis-, migration-, invasion-, and immune-related parameters [77,112]. Taken together, these findings suggest that AR- and AMR-derived constituents may influence several biological processes relevant to HCC, including cell-cycle regulation, programmed-cell-death-related pathways, redox balance, migration and invasion, and immune-related signaling (Figure 5).

However, existing evidence regarding hepatocellular carcinoma (HCC) should be interpreted with caution. Most studies involve cancer cell lines, network pharmacology predictions, or preclinical tumor models, and there is a lack of evidence from patients. Predicted compound-target networks cannot establish direct molecular interactions, and cytotoxic or anti-migration effects observed at experimental concentrations do not prove that equivalent pharmacological activity can be safely achieved in humans. Furthermore, findings derived from single cell lines or xenograft models may not fully reflect the molecular and immunological heterogeneity of human HCC. Therefore, while these data demonstrate preclinical antitumor activity and generate mechanistic hypotheses, they do not establish therapeutic efficacy in HCC patients.

## 9. Pharmacokinetics

Pharmacokinetic research on the active components of medicinal plants in the genus *Atractylodes* has made some progress. Currently, this research primarily focuses on representative compounds such as atractylodin, atractylon, and atractylenolides, covering processes such as absorption, tissue distribution, and metabolism. Atractylodin is a characteristic component of AR. Existing studies indicate that after oral administration, atractylodin is absorbed relatively quickly, but its systemic exposure and residence time in the body are relatively limited [113]. Following gastric administration of a Pingwei San suspension to rats, the time to peak concentration (T_max) of atractylodin was 2.5 h, the maximum plasma concentration (C_max) was 1.765 mg/L, the elimination half-life (t__1_/_2_z) was 5.723 h, and AUC was 11.789 mg·h/L; a second peak appeared on the plasma concentration-time curve approximately 6 h after administration, suggesting that its pharmacokinetics may involve mechanisms such as the enterohepatic circulation or gastrointestinal reabsorption [114]. Studies on tissue distribution have shown that, following oral administration, atractylodin is distributed to tissues such as the heart, liver, spleen, lungs, kidneys, and the gastrointestinal tract, with the highest concentrations found in the stomach and small intestine [115]. In a pharmacokinetic study of Rikkunshito, following oral administration of 7.5 g to healthy subjects, the geometric mean C_max of atractylodin was 1570 pg/mL, with a T_max of approximately 0.5 h, suggesting that it is also rapidly absorbed in humans [116]. Phase I pharmacokinetic studies in humans also showed that, following oral administration of a standardized AR extract, atractylodin enters the systemic circulation, and no significant accumulation was observed after 21 days of continuous administration [117].

Atractylon is a major component of the essential oil of both AR and AMR, though its relative abundance varies between species. Previous studies have established a quantitative analytical method for atractylon in rat plasma using gas chromatography–mass spectrometry (GC–MS). This method exhibits good linearity in the range of 10–1000 ng/mL, with a limit of quantification of 10 ng/mL, and has been successfully applied to pharmacokinetic studies following oral administration of *Atractylodes* extract. Following oral administration of *Atractylodes* extract to rats, the T_max of atractylon was 1.83 ± 0.41 h, the C_max was 257.1 ± 125.1 ng/mL, and the t__1_/_2_z was 7.64 ± 3.42 h [118]. Furthermore, atractylon was detected in the plasma of rats following gavage with the essential oil of Yinchen Shufu Decoction, indicating that it can be absorbed through the gastrointestinal tract and enter the systemic circulation [119,120]. However, compared with atractylodin and atractylenolides, the systematic studies on the absorption, distribution and metabolism of atractylon are still obviously insufficient.

Atractylenolides are representative constituents of AMR, and the pharmacokinetic studies of atractylenolides I, II, and III have been relatively well-documented. Atractylenolide I is rapidly absorbed following oral administration. As determined by capillary gas chromatography–selective ion monitoring mass spectrometry, the T_max in rats was 0.37 ± 0.19 h, the C_max was 0.26 ± 0.05 μg/mL, and the AUC was 1.95 ± 0.30 μg·h/mL [121]. Another LC–MS/MS study reported a T_max of 0.81 ± 0.11 h, a C_max of 7.99 ± 1.20 ng/mL, and a t__1_/_2_z of 1.94 ± 0.27 h [122]. There are significant differences in the parameters reported across different studies, which may be related to variations in dosage, dosage form, and analytical methods; therefore, direct numerical comparisons should be avoided. Tissue distribution studies showed that, following oral administration of 50.0 mg/kg Atractylenolide I to rats, the overall tissue concentrations followed the order: liver > kidney > spleen > cerebellum > heart > brain > lung, suggesting that the liver and kidneys may be the primary organs of distribution and metabolism. Its protein binding rates in rat plasma, human plasma, and bovine serum albumin were 80.8 ± 3.9%, 90.6 ± 3.1%, and 60.9 ± 5.1%, respectively, indicating a high degree of plasma protein binding [121,123]. Atractylenolide II is also rapidly absorbed from the gastrointestinal tract, and its primary site of intestinal absorption is believed to be the duodenum [124]. A UPLC-MS/MS method has been used for the quantification of atractylenolide II in rat plasma, with a linear range of 4–500 ng/mL [125]. Atractylenolide III also exhibits rapid oral absorption. A study showed that after oral administration of atractylenolide III to male SD rats, T_max was 45 min, t__1_/_2_z was 172.1 min, C_max was 1211 ng/L, and AUC was 156,031 ng∙min/L, further supporting its rapid absorption profile [126]. In addition, Atractylenolide III undergoes various metabolic reactions in vivo, including oxidation, hydroxylation, methylation, glucuronidation, and sulfation. Previous studies have identified more than 50 related metabolites in rat plasma, urine, and feces, indicating that it undergoes a relatively complex metabolic process in vivo [127]. Hepatic microsome studies further indicate that Atractylenolide III may exhibit sex-related metabolic differences; the residual amounts after incubation with hepatic microsomes from male and female rats were approximately 27% and 57%, respectively, suggesting that its metabolic stability may be sex-dependent [128].

It is worth noting that processing methods and compound formulations can further alter the pharmacokinetic behavior of active components in *Atractylodes*. Following administration of bran-fried AR, both the absorption rate and systemic exposure of atractylodin increase [129]. UPLC-MS/MS studies have also shown that, compared with raw AR, the combined C_max and AUC of atractylenolide I, II, and III, as well as atractyloside A, are elevated following administration of bran-fried AR, suggesting that bran-fried AR not only alters the chemical composition of the herbal material but may also modify its overall in vivo exposure by influencing the dissolution, absorption, and interactions of its constituents [125,130]. At the same time, pharmacokinetic parameters for the same constituent may vary significantly depending on the specific formulation, indicating that constituent-constituent interactions within complex traditional Chinese medicine systems are important factors influencing the in vivo processes of *Atractylodes* species [131].

Overall, there are significant differences in the pharmacokinetic characteristics of the major active components of *Atractylodes*. Atractylodin and atractylenolides are generally rapidly absorbed after oral administration; however, some components exhibit high plasma protein binding, complex tissue distribution, and biphasic plasma concentration-time curves. In contrast, systemic pharmacokinetic data on atractylon remain relatively scarce. It is worth noting that the existing evidence is primarily derived from animal studies, and there are considerable differences in dosage, administration methods, and analytical techniques across different studies, which limits direct comparison of parameters. More importantly, the doses and formulations used in many pharmacological studies are not directly comparable with those evaluated in available pharmacokinetic studies. Consequently, the current evidence does not establish whether the concentrations associated with biological activity in experimental models can be achieved and maintained safely in humans. This exposure–effect gap is particularly relevant when interpreting studies using purified compounds at relatively high experimental doses or polysaccharide preparations with limited systemic bioavailability. To address these challenges, future research should conduct standardized absorption, distribution, metabolism, and excretion (ADME) studies to determine the absolute bioavailability and metabolite profiles of the major active ingredients. In parallel, human pharmacokinetic investigations and simultaneous multi-component pharmacokinetic analyses of complex formulations should be strengthened to establish a clearer relationship between the chemical constituents of *Atractylodes*, in vivo exposure, and pharmacological effects.

## 10. Toxicity

In vivo studies using animal models, including mice, rats, and zebrafish embryos, have evaluated the safety and toxicity profiles of AR and AMR in preclinical settings. Acute oral toxicity studies of crude ethanol extracts of AR (*A. lancea*) demonstrated that the median lethal dose (LD_50_) in mice was greater than 2000 mg/kg, with no obvious hepatic, renal, or other organ pathological alterations observed [132]. In addition, ethanol extracts of AR (*A. chinensis*) were shown to alleviate inflammatory responses through inhibition of the NF-κB signaling pathway at therapeutic doses, without inducing apparent toxic effects [133]. Similar safety profiles have been reported for AMR. Acute oral toxicity studies in rats and mice using the maximum dose method showed that the LD_50_ of AMR extracts was ≥10 g/kg body weight, and no signs of toxicity or mortality were observed at doses of 10 g/kg or higher. Furthermore, in a 90-day subchronic toxicity study, rats were continuously fed diets containing 5.0%, 2.5%, or 1.0% AMR extract. No significant toxicological changes were observed in clinical signs, body weight, hematological parameters, serum biochemical indicators, or histopathological examination of major organs, indicating no overt toxicity under the tested preclinical conditions [134].

Nevertheless, despite the relatively broad safety margins of crude herbal materials, studies focusing on major bioactive constituents have revealed dose-dependent toxicological effects that warrant attention. Zebrafish embryo models demonstrated that exposure to atractylenolides I, II, and III resulted in concentration-dependent mortality, with 96 h median lethal concentrations (LC_50_) of 81.64 μM, 138.40 μM, and 151.90 μM, respectively [135]. Atractylenolides I and II induced significant developmental abnormalities and hepatotoxicity in zebrafish embryos, characterized by hepatic atrophy, reduced liver-specific fluorescence intensity, increased AST and ALT levels, increased apoptosis, and inhibition of drug-metabolizing enzyme activity; in contrast, exposure to atractylenolide III did not induce significant toxic effects. Furthermore, at specific concentrations, β-eudesmol and atractylon were associated with reduced embryo survival rates, developmental abnormalities, and altered expression of oxidative stress-related genes, suggesting potential risks of embryotoxicity and oxidative injury [136].

Collectively, available preclinical toxicological studies suggest that crude AR and AMR preparations may be tolerated under some of the tested experimental conditions, whereas individual bioactive constituents can exhibit concentration-dependent toxic effects. In particular, atractylenolides I and II have been associated with developmental abnormalities and hepatotoxicity in zebrafish embryos, while β-eudesmol and atractylon have also shown potentially unfavorable effects at specific experimental concentrations [135,136]. These findings cannot be used to define clinically relevant therapeutic windows or safe dosing regimens in humans. Human long-term safety, reproductive and developmental toxicity, herb–drug interactions, and safety in vulnerable populations remain insufficiently characterized. Systematic toxicological and clinical safety studies are therefore required before conclusions regarding the safety of long-term or therapeutic use of individual *Atractylodes*-derived compounds can be established.

## 11. Limitations

Despite the increasing evidence supporting the pharmacological activities of *Atractylodes* medicinal plants in liver diseases, several important limitations should be considered when interpreting the current findings [137]. Although numerous studies have demonstrated that *Atractylodes*-derived compounds exert protective effects against metabolic disorders, oxidative stress, inflammatory responses, fibrosis progression, and tumor development, most available evidence is still derived from in vitro studies and animal models rather than high-quality clinical investigations [138]. Therefore, the current understanding of their therapeutic potential should be interpreted with caution. A major unresolved issue is whether the biological effects induced by *Atractylodes*-derived compounds result from direct interactions with specific molecular targets or represent secondary consequences of broader cellular regulatory events. Previous studies have reported that atractylodin, atractylon, and atractylenolides can modulate multiple signaling pathways, including AMPK, Nrf2, NF-κB, and TGF-β/Smad; however, alterations in signaling pathways alone do not provide sufficient evidence for direct target engagement [9,11,12]. Most current studies rely primarily on changes in protein expression levels, phosphorylation status, or pharmacological inhibitor experiments, whereas direct validation of compound–protein interactions remains insufficient [139]. Therefore, future studies integrating chemical biology, structural biology, and proteomic approaches are required to identify precise molecular targets and establish reliable structure–activity relationships.

In addition to small-molecule constituents, the pharmacological mechanisms of *Atractylodes*-derived polysaccharides remain incompletely understood. Polysaccharides obtained from AR and AMR have demonstrated hepatoprotective effects in various experimental models; however, their high molecular weight and hydrophilic characteristics generally result in limited intestinal absorption and relatively low systemic bioavailability [44,140]. Current evidence suggests that these polysaccharides may exert biological effects primarily through modulation of intestinal barrier integrity, gut microbiota composition, and microbiota-derived metabolites via the gut–liver axis rather than through direct hepatic exposure [72]. Nevertheless, direct evidence distinguishing local intestinal effects from systemic actions remains insufficient. Furthermore, although AR and AMR share several bioactive constituents and both exhibit potential hepatoprotective effects, they possess distinct chemical profiles, with AR being enriched in polyacetylenes and AMR containing higher levels of sesquiterpene lactones. These chemical differences suggest that the two medicinal materials may possess different pharmacological characteristics; however, systematic comparative studies using standardized extracts, identical disease models, and unified evaluation criteria are currently lacking. Consequently, evidence-based selection of AR or AMR for specific liver disease indications has not yet been established.

Methodological heterogeneity among existing studies further limits the interpretation, comparison, and clinical translation of current findings. Differences in animal species, genetic background, disease induction protocols, extraction procedures, compound purity, administration routes, and dosage regimens represent major sources of variability among studies [141]. Moreover, many investigations lack comprehensive dose–response analyses, clinically relevant positive controls, long-term intervention designs, and independent validation experiments. Since chronic liver diseases usually develop over years in humans, short-term experimental models may not adequately reflect long-term therapeutic efficacy and safety. Importantly, clinical evidence regarding *Atractylodes*-derived compounds remains extremely limited. Most available human studies involve multi-component herbal formulations rather than isolated active compounds, making it difficult to determine compound-specific clinical efficacy. Therefore, well-designed randomized controlled trials, pharmacokinetic studies, and biomarker-guided clinical investigations are urgently needed to determine whether promising preclinical findings can be translated into clinically meaningful therapeutic strategies. Safety evaluation also remains a critical concern. Although *Atractylodes* medicinal materials have a long history of traditional use and are generally considered safe at conventional doses, systematic toxicological evaluation of purified bioactive compounds remains insufficient. Their therapeutic windows, long-term toxicity profiles, potential herb–drug interactions, and safety in specific populations require further investigation.

Furthermore, inconsistencies among published studies highlight the urgent need for standardized research frameworks. For example, different studies have reported apparently divergent regulatory effects of *Atractylodes*-derived components on autophagy. Atractylenolide I has been reported to promote autophagic activity in acute-on-chronic liver failure models, whereas *Atractylodes* polysaccharides have been shown to suppress excessive autophagy in metabolic liver disease models [10,76]. These differences may reflect disease-stage specificity, compound-dependent mechanisms, or variations in experimental conditions rather than genuine mechanistic contradictions. Similarly, differences in polysaccharide preparation methods, structural characteristics, and administration doses contribute to variability in pharmacological outcomes. Overall, current evidence supports the potential pharmacological activities of *Atractylodes* medicinal plants; however, definitive clinical efficacy has not yet been established. Future research should prioritize molecular target identification, pharmacokinetic characterization, standardized preparation development, comprehensive safety evaluation, and rigorously designed clinical trials to facilitate the transition of *Atractylodes*-derived compounds from experimental observations toward evidence-based therapeutic applications.

## 12. Conclusions and Outlook

Current evidence regarding *Atractylodes* medicinal plants and their bioactive constituents in liver diseases is predominantly derived from in vitro experiments and animal models. Across these studies, *Atractylodes*-derived extracts, polysaccharides, and small-molecule constituents have been associated with changes in lipid metabolism, oxidative-stress-related responses, inflammatory signaling, gut–liver axis-related parameters, hepatic stellate cell activation, and tumor-cell phenotypes. Collectively, these findings support continued investigation of *Atractylodes*-derived preparations and constituents in experimental models of liver disease. However, the available data do not establish clinical therapeutic efficacy, and many of the proposed mechanisms remain based on pathway-associated changes rather than direct molecular target validation.

**Figure 6:**
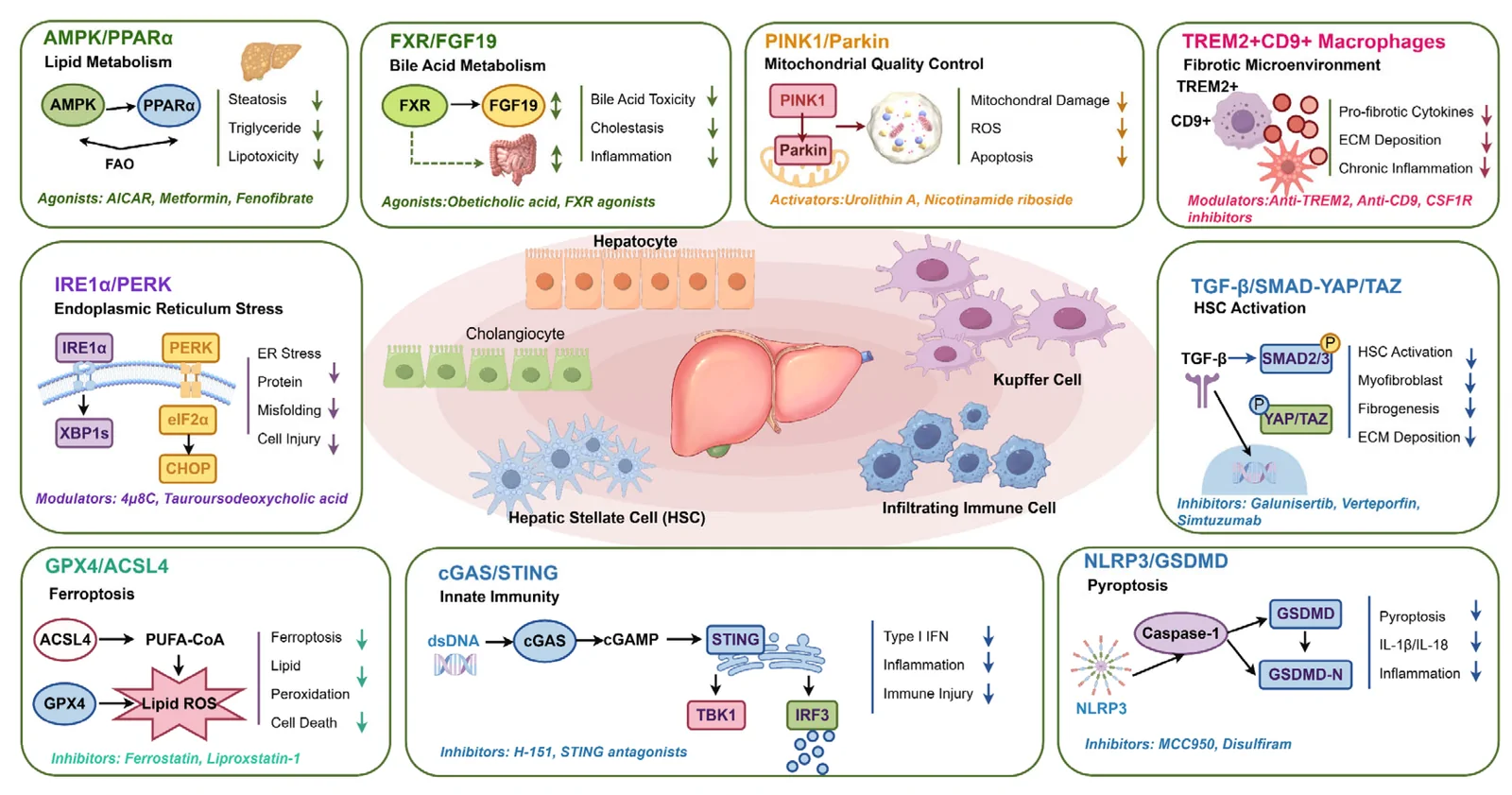
Emerging mechanisms for intervening in liver disease. Note: References [142,143,144,145,146,147,148,149,150]. Evidence base: The evidence summarized in this figure is derived from in vitro studies and in vivo animal models. Abbreviations: ACSL4, acyl-CoA synthetase long-chain family member 4; AICAR, 5-aminoimidazole-4-carboxamide ribonucleotide; AMPK, AMP-activated protein kinase; CD9, cluster of differentiation 9; cGAMP, cyclic guanosine monophosphate–adenosine monophosphate; cGAS, cyclic GMP–AMP synthase; CHOP, C/EBP homologous protein; CSF1R, colony-stimulating factor 1 receptor; dsDNA, double-stranded DNA; ECM, extracellular matrix; eIF2α, eukaryotic translation initiation factor 2 alpha; ER, endoplasmic reticulum; FAO, fatty acid oxidation; FGF19, fibroblast growth factor 19; FXR, farnesoid X receptor; GPX4, glutathione peroxidase 4; GSDMD, gasdermin D; GSDMD-N, N-terminal fragment of gasdermin D; HSC, hepatic stellate cell; IFN, interferon; IL, interleukin; IRE1α, inositol-requiring enzyme 1 alpha; IRF3, interferon regulatory factor 3; NLRP3, NOD-like receptor family pyrin domain-containing 3; PERK, protein kinase R-like endoplasmic reticulum kinase; PINK1, PTEN-induced kinase 1; PPARα, peroxisome proliferator-activated receptor alpha; PUFA-CoA, polyunsaturated fatty acyl-CoA; ROS, reactive oxygen species; SMAD2/3, SMAD family members 2 and 3; STING, stimulator of interferon genes; TAZ, transcriptional coactivator with PDZ-binding motif; TBK1, TANK-binding kinase 1; TGF-β, transforming growth factor beta; TREM2, triggering receptor expressed on myeloid cells 2; XBP1s, spliced X-box binding protein 1; YAP, Yes-associated protein. The arrows in this figure indicate proposed relationships or signaling-related changes rather than established causal mechanisms.

In recent years, research on liver diseases has increasingly focused on molecular and cellular processes that may provide targets for mechanism-based therapeutic interventions (Figure 6). Representative molecular and cellular processes currently investigated as potential intervention targets include AMPK/PPARα-mediated lipid metabolism [142], FXR/FGF19-regulated bile acid homeostasis [143], PINK1/Parkin-dependent mitochondrial quality control [144], IRE1α/PERK pathway-mediated endoplasmic reticulum stress [145], GPX4/ACSL4-regulated ferroptosis [146], NLRP3/GSDMD-mediated pyroptosis [147], cGAS/STING-associated innate immune activation [148], the fibrotic microenvironment shaped by TREM2^+^CD9^+^ scar-associated macrophages [149], and TGF-β/Smad–YAP/TAZ-related hepatic stellate cell activation [150]. These emerging mechanisms provide a broader framework for identifying future research directions for *Atractylodes*-derived compounds. Future studies should determine which pathways, if any, are directly and reproducibly modulated by *Atractylodes*-derived compounds under pharmacologically relevant exposure conditions. Particular priorities include direct target validation, standardized preparations and disease models, dose–response assessment, independent replication, and integrated pharmacokinetic–pharmacodynamic studies.

In addition, important translational uncertainties remain. The relationship between experimentally active doses and achievable human exposure is insufficiently defined, while clinically relevant therapeutic windows, long-term safety, herb–drug interactions, and safety in specific populations require systematic evaluation. Clinical evidence is also limited and frequently involves multi-component herbal formulations, making compound-specific efficacy difficult to determine. Overall, the available evidence provides a preclinical pharmacological rationale for further investigation of *Atractylodes* and its bioactive constituents in liver diseases. However, their direct molecular targets, clinically relevant pharmacokinetics, long-term safety, and therapeutic efficacy in humans remain to be established through rigorous mechanistic, translational, and clinical studies.

## Figures and Tables

**Table 1:** Comparison of the present review with previous representative reviews.

Dimension	Unique Contributions of This Review	Previous Reviews	References
Botanical scope	Three major species of the genus *Atractylodes* (*A. lancea*, *A. chinensis*, *A. macrocephala*)	One or two species of the genus *Atractylodes*	[5,6,7,8]
Coverage of liver diseases and pathological contexts	MASLD and ALD; hepatic fibrosis and cirrhosis; and HCC	Focused on a single disease type or providing only a general overview	[6,8]
Mechanistic depth	Stage-specific regulatory networks governing metabolism, inflammation, oxidative stress, autophagy, fibrosis, and tumorigenesis	Provides only a general overview, with limited detail on the temporal and pathological progression of liver disease	[15]
Pharmacokinetics and safety	Dedicated section with ADME and toxicity	Limited coverage	[15,16]
Critical gaps and future directions	Candidly addresses limitations: Lack of direct target binding data, unclear safety margins, scarce high-quality clinical trials, and the unresolved direct vs. indirect action of polysaccharides	Often present a more descriptive summary without critically evaluating translational bottlenecks or safety concerns	[8,15]

## Data Availability

No new data were created or analyzed in this study. Data sharing is not applicable to this article.

## Abbreviations

Major abbreviations used in this manuscript:

ACAT	Acyl-CoA cholesterol acyltransferase
ACLF	Acute-on-chronic liver failure
ALD	Alcohol-associated liver disease
ALT	Alanine aminotransferase
AMPK	AMP-activated protein kinase
Apo-A1	Apolipoprotein A1
AST	Aspartate aminotransferase
CAT	Catalase
ceRNA	Competing endogenous RNA
CYP2E1	Cytochrome P450 2E1
ECM	Extracellular matrix
EMT	Epithelial–mesenchymal transition
ERK	Extracellular signal-regulated kinase
FASN	Fatty acid synthase
FTH1	Ferritin heavy chain 1
GPX4	Glutathione peroxidase 4
GSH	Glutathione
GSH-Px	Glutathione peroxidase
HCC	Hepatocellular carcinoma
HDL	High-density lipoprotein
HO-1	Heme oxygenase-1
HSC	Hepatic stellate cell
JNK	c-Jun N-terminal kinase
LCAT	Lecithin-cholesterol acyltransferase
LPS	Lipopolysaccharide
LXR	Liver X receptor
MASLD	Metabolic dysfunction-associated steatotic liver disease
MAPK	Mitogen-activated protein kinase
MASH	Metabolic dysfunction-associated steatohepatitis
MDA	Malondialdehyde
MMP	Matrix metalloproteinase
mTOR	Mammalian target of rapamycin
MyD88	Myeloid differentiation primary response 88
NAFLD	Non-alcoholic fatty liver disease
NASH	Non-alcoholic steatohepatitis
NF-κB	Nuclear factor kappa-B
NLRP3	NOD-like receptor family pyrin domain containing 3
NOX4	NADPH oxidase 4
Nrf2	Nuclear factor erythroid 2-related factor 2
PPAR	Peroxisome proliferator-activated receptor
RCT	Reverse cholesterol transport
ROS	Reactive oxygen species
SIRT1	Sirtuin 1
SLC7A11	Solute carrier family 7 member 11
Smad	Mothers against decapentaplegic homolog
SOD	Superoxide dismutase
SR-BI	Scavenger receptor class B type I
SREBP-1c	Sterol regulatory element-binding protein 1c
TAZ	Transcriptional coactivator with PDZ-binding motif
TGF-β1	Transforming growth factor beta 1
TLR4	Toll-like receptor 4
YAP	Yes-associated protein
AR	Atractylodis Rhizoma
AMR	Atractylodis Macrocephalae Rhizoma
